# Identification of suitable reference genes for gene expression studies by qRT-PCR in the blister beetle *Mylabris cichorii*

**DOI:** 10.1093/jis/14.1.94

**Published:** 2014-07-18

**Authors:** Yu Wang, Zhong-Kang Wang, Yi Huang, Yu-Feng Liao, You-Ping Yin, Jake Tu

**Affiliations:** 1 Bio-Engineering College of Chongqing University, Key Lab of Genetic Function and Regulation in Chongqing, Chongqing, China; 2 Panzhihua University, Panzhihua, China

**Keywords:** cantharidin, normalization

## Abstract

The blister beetle *Mylabris cichorii* L. (Coleoptera: Meloidae) is a traditional medicinal insect recorded in the Chinese Pharmacopoeia. It synthesizes cantharidin, which kills cancer cells efficiently. Only males produce large amounts of cantharidin. Reference genes are required as endogenous controls for the analysis of differential gene expression in *M. cichorii* . Our study chose 10 genes as candidate reference genes. The stability of expression of these genes was analyzed by quantitative PCR and determined with two algorithms, geNorm and Normfinder. We recommend UBE3A and RPL22e as suitable reference genes in females and UBE3A, TAF5, and RPL22e in males.

## Introduction

Blister beetles, the common name of Meloidae insects, have been used in Europe to treat diseases such as rabies, dropsy, warts, impotence, and fever ( Moed et al. 2001 ). *Mylabris cichorii* L. (Coleoptera: Meloidae) is a traditional Chinese medicinal insect recorded in Chinese Pharmacopoeia. The dried bodies of these insects have been used as a traditional Chinese medicine for more than 2000 years ( Wang 1989 ). Cantharidin can be produced by all coleopteran species as a defensive secretion when the adult beetles are attacked ( Carrel et al. 1993 ). It has been found that cantharidin and its derivatives play significant roles in the therapy of several cancers, such as liver cancer, esophageal cancer, and stomach cancer ( Yang et al. 2007 ; Liu and Chen 2009 ). In addition, cantharidin has also been identified as a potential insecticide to control agricultural pests such as *Mythimna separata* and *Plutella xylostella* L. ( Carrel and Eisner 1974 ; Zhang et al. 2003 ).

The biological studies of Meloidae insects have shown that the adults have sexual dimorphism in cantharidin production. Cantharidin is mostly synthesized by male beetles and then transferred to the female as a precopulatory gift ( Eisner et al. 1996 ; Wang et al. 2008 ), but it is still not clear how and where the cantharidin is synthesized in meloid beetles ( Sierra et al. 1976 ; Carrel et al. 1993 ; Nikbakhtzadeh et al. 2007 ). Previous research showed that male *M. cichorii* had very low levels of cantharidin after emergence, but the amount increased rapidly after 25 days and reached its peak at the 30 day in the male breeding group. In the female breeding group, the amount of cantharidin remained at a low level ( Wang et al. 2008 ). To study the pathway of cantharidin biosynthesis, it is necessary to investigate the differentially expressed genes between adult females and males.

Real-time reverse transcription quantitative PCR (qRT-PCR) has been widely used in the expression profiling of selected genes in biological research. Normalization is critically important to correct for inter-sample variations caused by differences in reaction efficiency or sample preparation. Suitable reference genes will enable more accurate normalization and quantification of gene expression. Unfortunately, the selection of suitable reference genes for normalization is often ignored in the gene expression studies of insects ( Scharlaken et al. 2008 ). Only a few insect’s reference genes have been validated and published, including the silkworm ( Wang et al. 2008 ), the honeybee ( Scharlaken et al. 2008 ), the fruit fly ( Shen et al. 2010 ), the red flour beetle ( Lordet al. 2010 ), the locust (Chapuis et al. 2011), the planthopper ( Maroniche et al. 2011 ), and *Liposcelis bostrychophila* ( Jiang et al. 2010 ).

The aim of our study is to identify the most stably expressed candidate reference genes for gene expression studies in the *M. cichorii* adult. The selected candidate reference genes were orthologs of the commonly used genes, including ribosomal proteins (RPL22e, RPL13a, RPS27), actin (ACT), P-TUB, ubiquitin C (UBC), ubiquitin-conjugating enzyme E2C (UBE2C), ubiquitin-protein ligase E3A (UBE3A), elongation factor-1 alpha (EF1A), and transcription initiation factor TFIID subunit 5 (TAF5). The reliability of selected candidate genes was validated by qRT-PCR.

## Materials and Methods

### Insects

The oriental *M. cichorii* was originally collected from Guizhou Province, China and reared in our laboratory. In order to ensure uniform development, adult beetles were discretely reared in the laboratory and fed on a semi-artificial diet under the stable photoperiod cycle of 14:10 L:D at 30 ± 1°C and 75 ± 5% RH ( Wang et al. 2008 ). Male and female adults were collected 5, 10, 15, 20, 25, and 30 days after emergence from the rearing population. The total RNA was extracted from the collected samples. Triplicates were run for the male and female groups (five insects per pooled sample).

### Isolation of total RNA and cDNA synthesis

The total RNA was extracted from collected adults at different developmental stages using TRIzol reagent (Invitrogen, www.lifetechnologies.com ). For genomic DNA removal, total RNA was treated with RNase-free DNase I (Fermentas, Thermo Scientific, www.thermoscientificbio.com ). Finally, the total RNA was dissolved in 40 µL RNase-free water and stored at -80°C for fur ther use. Yield of RNA was assessed by the absorbance at 260 nm using a Du® Series 640 Spectrophotometer (Beckman Coulter, www.beckmancoulter.com ). The purity of RNA was determined at an absorbance ratio of OD260/280, and the integrity was checked by 1% agarose gel electrophoresis.

The first strand cDNA was synthesized from 2 µg of DNA-free RNA using the RevertAid™ First Strand cDNA synthesis kit (Fermentas) for qRT-PCR. After the reverse transcription, the synthesized cDNA was stored at -20°C for further use.

### Candidate reference genes and their quantitative real-time PCR

In this study, we selected ten candidate reference genes, RPL22e, RPL13a, RPS27, ACT, β-TUB, UBC, UBE2C, UBE3A, EF1A, and TAF5. The primers for amplification of the reference genes were designed using Primer 5.0 (Premier Biosoft, www.premierbiosoft.com ). Ten-fold serial dilutions of pooled cDNA samples were used to construct a relative standard curve for determining PCR efficiency (E%) of each primer pair for each gene. qRT-PCR was carried out in an optical 96-well plate with a MyCyclerTM Thermal Cycler system (Bio-Rad, www.bio-rad.com ). SYBR Premix Ex Taq II (Clontech, www.clontech.com ) was used to monitor dsDNA synthesis. In a total volume of 25 µL, 12.5 µL of SYBR® Premix Ex TaqTM II (2×) was mixed together with 0.5 µL of the PCR forward primer (10 µM), 0.5 µL of the PCR reverse primer (10 µM), 1 µL of cDNA, and 10.5 µL of sterile water. The qRT-PCR reaction was run at 95°C for 10 min (pre-denaturation) followed by 42 cycles at 95°C for 15 sec, at 58°C for 30 sec, and at 72°C for 30 sec. After reaction, a melting curve analysis from 55°C to 95°C was applied to all reactions to ensure consistency and specificity of the amplified product. The data were analyzed using the iQ ^TM^ 5 optical system software (Bio-Rad).

### Determination of reference gene expression stability

In order to compare the expression stability of the selected reference genes at different ages, the average Ct-value of each triplicate reaction were calculated for geNorm ( Vandesompele et al. 2002 ) and Normfinder programs ( Andersen et al. 2004 ) according to the developer’s instructions.

## Results

### Primers

The primers were designed and tested for 10 candidate reference genes (RPL22e, RPL13a, RPS27, ACT, β -TUB, UBC, UBE2C, UBE3A, EF1A, and TAF5). For each pair of primers, a dissociation curve with a single peak ensured that the primers amplified the unique product. All the tested primer sets showed the coefficient (R ^2^ ) was above 0.99, and the PCR efficiency (E%) of the 10 candidate reference genes ranged from 90.1 to 103.1 ( Table 1 ).

**Table 1.**
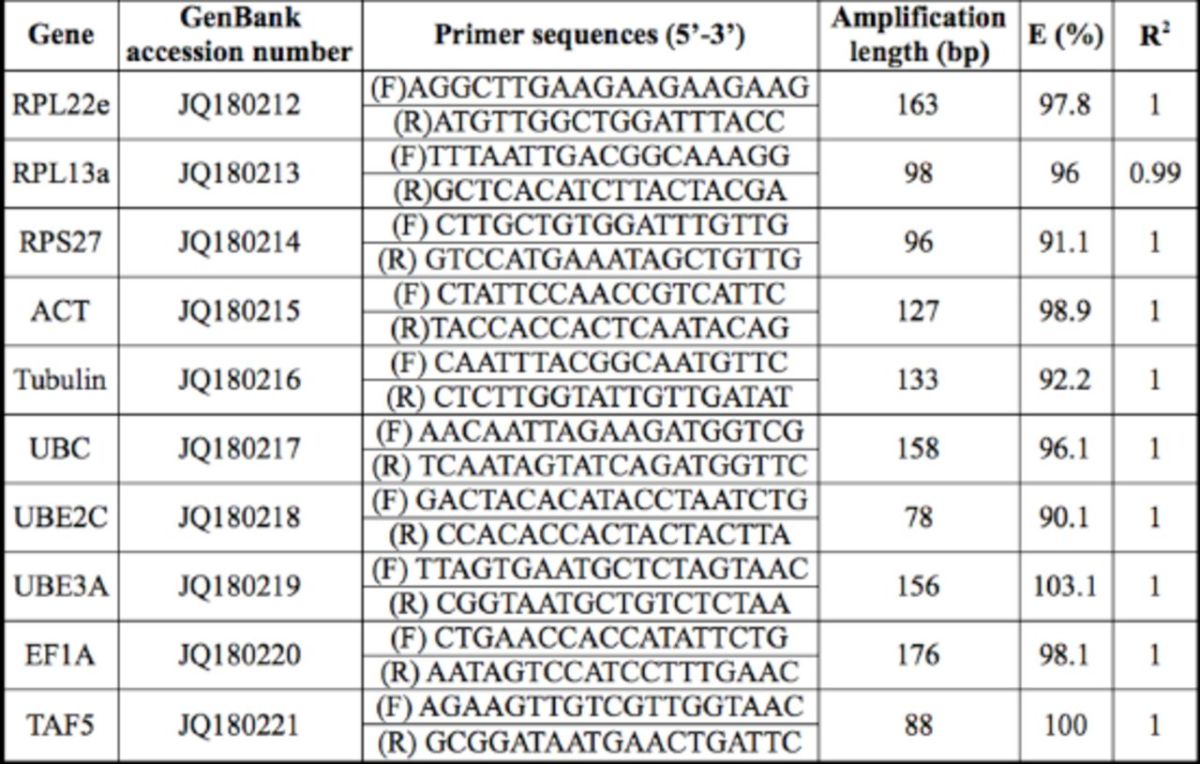
The primer pairs used for qRT-PCR.

E: PCR efficiency; R ^2^ : coefficient of determination

### Expression stability analysis of the candidate genes in adult femals

**Figure 1.**
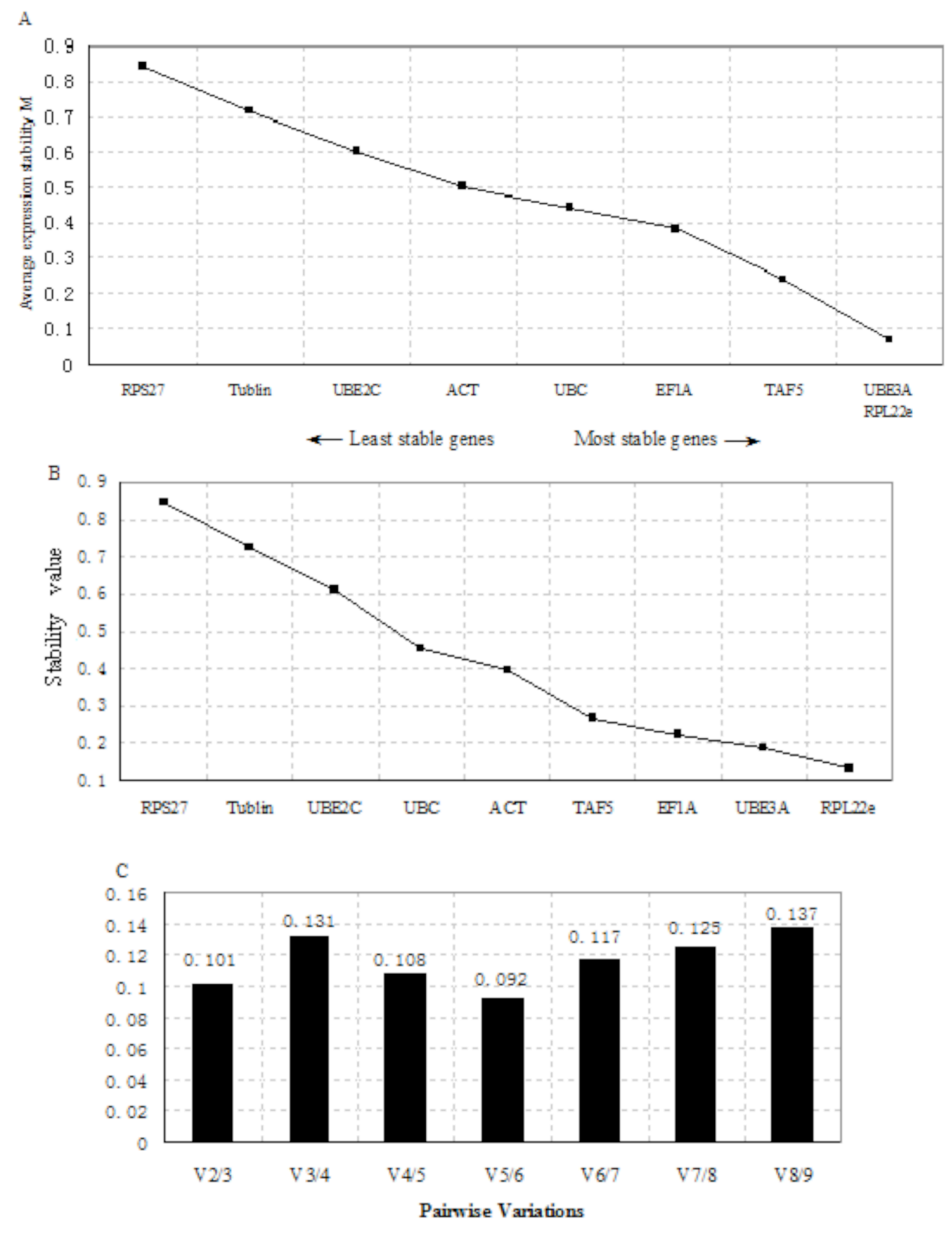
Ranking, stability, and determination of the optimal number of reference genes for female adult beetles. Expression stability of the candidate reference genes was analyzed by the geNorm program and Normfinder program. (A) geNorm gives an average expression stability measure (AESM) as the mean of the stability values of the remaining genes in a stepwise exclusion process. AESM of the control genes is plotted from least stable (left) to most stable (right). The two most stable genes are shown by a same M value in the right of the graph. (B) Normfinder also gave a stability value, and genes were ordered from least (left) to most (right). (C) Pairwise variation (Vn/n+1) analysis between the normalization factors NFn and NFn+1 determines the optimal number of reference genes for normalization. High quality figures are available online.

Because the expression patterns of RPL22e and RPL13a are similar, RPL22e was chosen for the calculation. The geNorm program was employed to estimate the expression stability of the candidate reference genes in adult fe-mals. According to their average expression stability (M value), the candidate reference genes were ranked from the most stable to the least ( Figure 1A ). It showed: UBE3A/ RPL22e < TAF5 < EF1A < UBC < ACT < UBE2C < Tubulin < RPS27. All candidate reference genes were below the default limit of M = 1.5. UBE3A and RPL22e showed the lowest M value (0.067) and they were the most stable genes, followed by TAF5. The ranking order of the candidate reference genes was shown by using the Normfinder program ( Figure 1B ). According to Normfinder, RPL22e was the best choice for a reference gene. UBE3A and EF1A were ranked as the second and third genes, respectively. In addition, the optimal number of reference genes for generating was considered. GeNorm compares the variation (V) between two sequential normalization factors. Because incorporating more genes has only a small effect on the newly calculated normalization factor, two or three reference genes would be sufficient to accurately normalize the data ( Vandesompele et al. 2002 ). The pairwise variation value V2/3 was less than V3/4 ( Figure 1C ). Thus, the result revealed that UBE3A and RPL22e were the most stable genes needed for a reliable normalization.

### Expression stability analysis of the candidate genes in male adult

**Figure 2.**
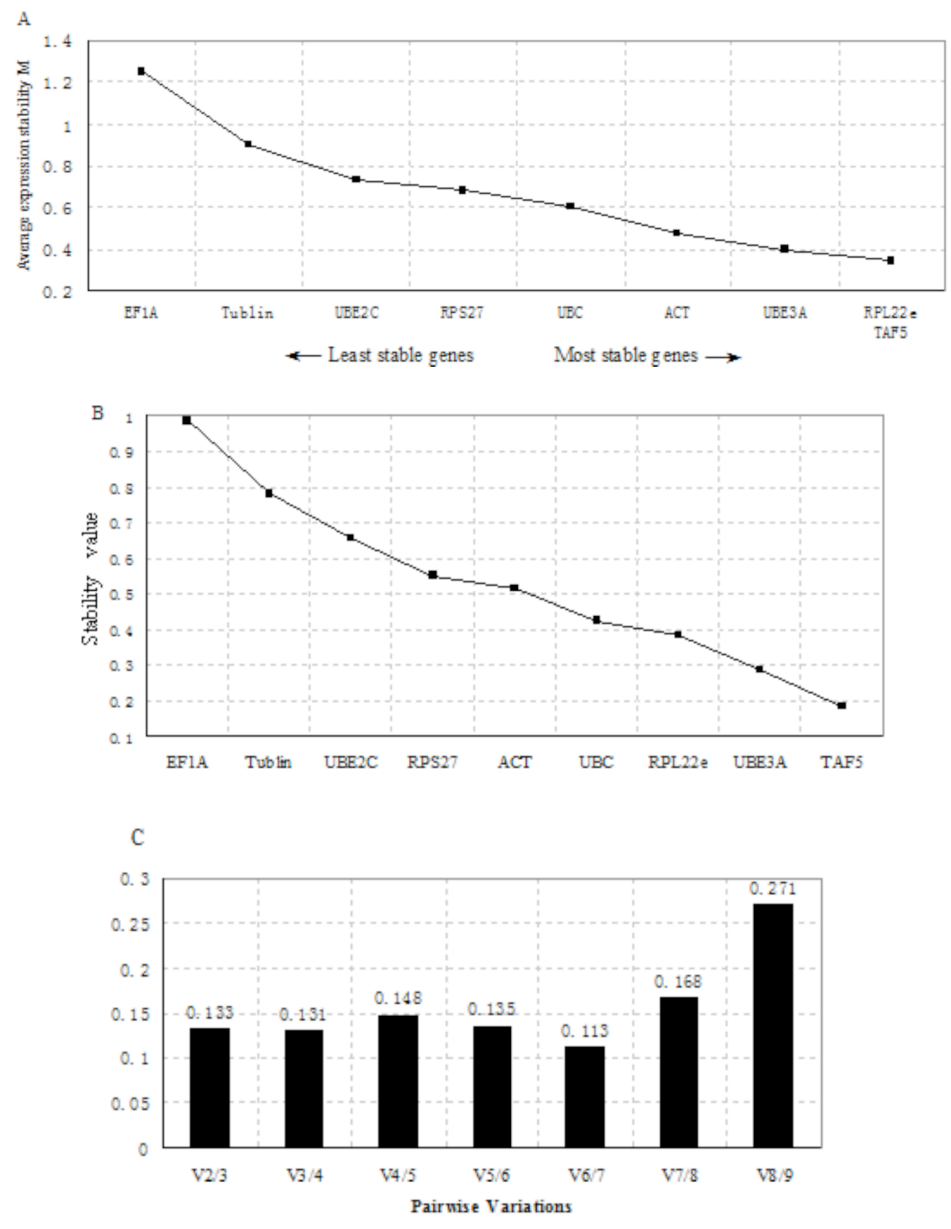
Ranking, stability, and determination of optimal number of reference genes for male adult beetles. Expression stability of the candidate reference genes was analyzed by the geNorm program and Normfinder program. (A) geNorm gives an AESM as the mean of the stability values of the remaining genes in a stepwise exclusion process. AESM of the control genes is plotted from least stable (left) to most stable (right). The two most stable genes are shown by a same M value in the right of the graph. (B) Normfinder also gave a stability value, and genes were ordered from least (left) to most (right). (C) Pairwise variation (Vn/n+1) analysis between the normalization factors NFn and NFn+1 determines the optimal number of reference genes for normalization. High quality figures are available online.

As shown in Figure 2A , the M values for the tested genes in adult males were below 1.5. Though the stability values for candidate reference gene transcripts in males were generally higher than in females, the expression of the genes was recognized as sufficiently stable. GeNorm indicated that RPL22e and TAF5 (AESM = 0.342) were the most stable genes, followed by UBE3A. According to Normfinder, TAF5 was the best choice for a reference gene in males. UBE3A and RPL22e were ranked as the second and third genes, respectively ( Figure 2B ). The pairwise variation values were also calculated by geNorm ( Figure 2C ). The result showed that the pairwise variation value V3/4 was less than V2/3. Based on all the above, the use of TAF5, UBE3A, and RPL22e as the stable reference genes is suggested.

### Expression stability analysis of the candidate genes in both female and male adults

**Figure 3.**
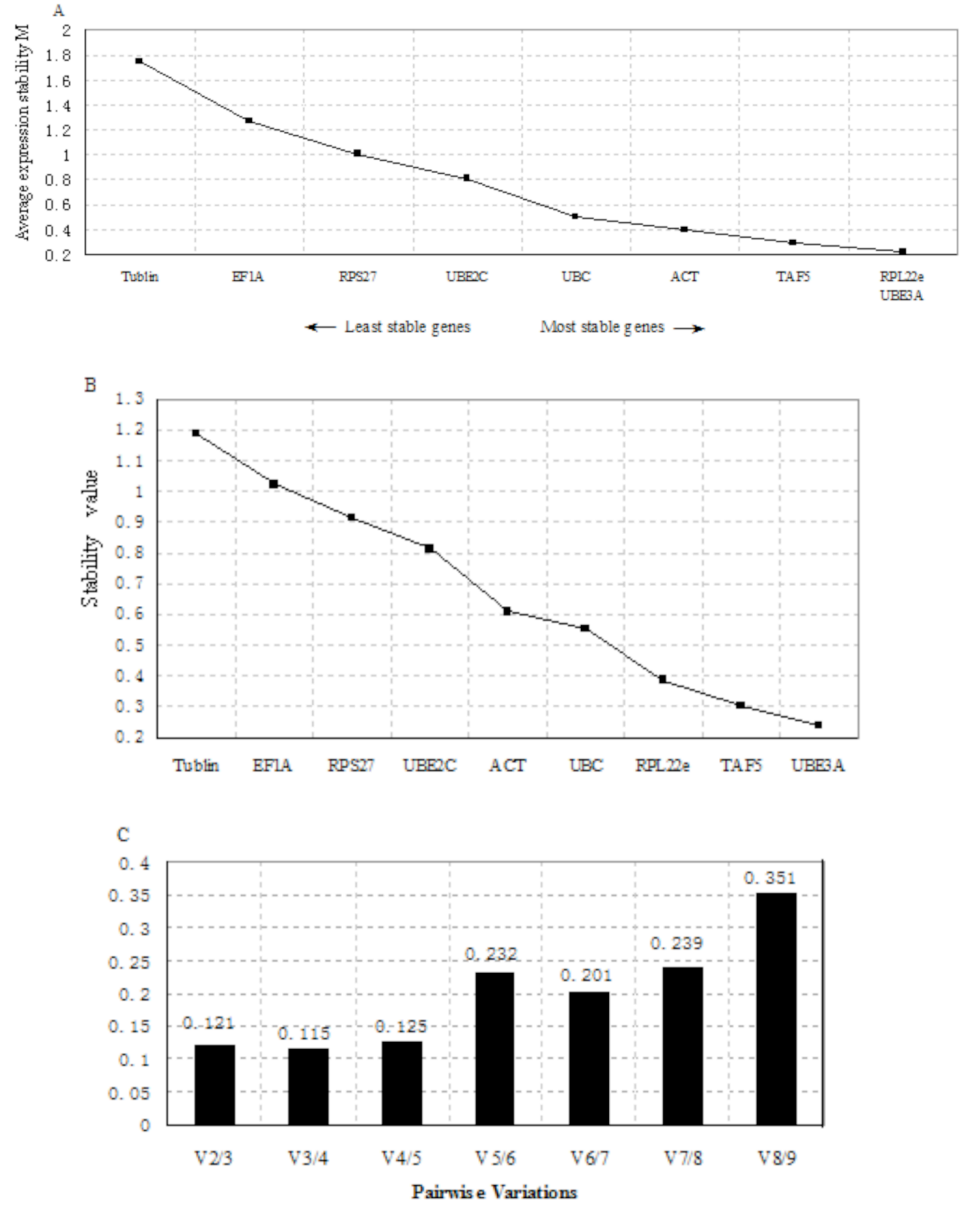
Ranking, stability, and determination of optimal number of reference genes for female and male adult beetles. Expression stability of the candidate reference genes was analyzed by the geNorm program and Normfinder program. (A) geNorm gives an AESM as the mean of the stability values of the remaining genes in a stepwise exclusion process. AESM of the control genes is plotted from least stable (left) to most stable (right). The two most stable genes are shown by a same M value in the right of the graph. (B) Normfinder also gave a stability value, and genes were ordered from least (left) to most (right). (C) Pairwise variation (Vn/n+1) analysis between the normalization factors NFn and NFn+1 determines the optimal number of reference genes for normalization. High quality figures are available online.

The candidate genes expressed in both female and male adult beetles were also analyzed together. UBE3A and RPL22e (AESM = 0.227) were ranked as the best reference genes by geNorm ( Figure 3A ), followed by TAF5. Normfinder indicated UBE3A as the best gene, followed by TAF5 and RPL22e ( Figure 3B ). GeNorm indicated that the optimal number of genes for accurate normalization was three ( Figure 3C ). So, UBE3A, RPL22e, and TAF5 were chosen as the most stable reference genes of the candidates tested.

## Discussion

The results showed that the sexual differences in gene expression quantification should be carefully considered in *M. cichorii.* The qRT-PCR analysis identified different combination of suitable reference genes for female adults and male adults. Moreover, the rankings of candidate genes between female and male adults were quite different. Previous studies demonstrated that the sexual differences should be considered when appropriate reference genes were selected for gene expression profiling in the oriental fruit fly ( Shen et al. 2010 ). A comprehensive understanding of sexual differentiation will help future studies of the pathway of cantharidin biosynthesis.

This is the first report detailing the identification of suitable reference genes for qRT-PCR analysis in *M. cichorii.* Combining the analysis of the two software programs, UBE3A, RPL22e, and TAF5 were identified as the most suitable reference genes. UBE3A plays a very important role in the protein modification process. RPL22e is a ribosomal protein gene that is involved in protein synthesis and translation. TAF5 is a transcription initiation factor that is involved in transcription and protein synthesis. Some translation factors, such as translation initiation factor 4A, translation initiation factor 3 subunit 4, and translation initiation factor 3 subunit 5, have been selected as reference genes in the silkworm ( Wang et al. 2008 ). The classic reference gene ACT, which has been reported to vary considerably in mammals and the silkworm (Selvey et al. 2001; Wang et al. 2008 ), exhibited moderate stability in our study. Unfortunately, another classic reference gene, P-TUB, was proved to be one of the most variably expressed genes at the adult stage. A similar result has been observed in *Tribolium castaneum,* which is another coleopteran ( Lord et al. 2010 ). Thus, it is crucial to validate these housekeeping genes prior to their use for normalization in qRT-PCR analysis.

GeNorm and NormFinder, the two most common software programs for assessing the appropriateness of reference genes, showed different results. GeNorm not only selects the optimal gene combination, but also determines the optimal number of reference genes. Normfinder is a model-based approach and calculates the stability values of the individual candidate reference genes for normalization ( Andersen et al. 2004 ). NormFinder allows for the determination of intra- and inter-group variation, while geNorm sequentially excludes the worst gene, ending up with two genes and ranking the genes with the degree of similarity of expression profile. Evidence has been shown that the use of a single gene for normalization led to relatively large errors,, while the use of multiple reference genes, belonging to different functional classes, can reduce the possibility of coregulation and generate more reliable results ( Vandesompele et al. 2002 ; Heckmann et al. 2006 ).

In conclusion, ours is the first study on systematic comparison of several conventional and potential reference genes in *M. cichorii* adults. By a combination of two software programs for data analysis, we recommend UBE3A and RPL22e as the suitable reference genes in female *M. cichorii* and UBE3A, TAF5, and RPL22e for male adults. These results will greatly benefit gene expression studies in *M. cichorii* .
